# Socio-Demographic Characteristics, Body Weight Status and Energy Intake among Users and Non-Users of Dietary Supplements among Government Employees in Putrajaya, Malaysia

**DOI:** 10.3390/nu13072248

**Published:** 2021-06-29

**Authors:** Muhamad Hasrol Mohd Ashri, Hazizi Abu Saad, Siti Nur Άsyura Adznam

**Affiliations:** 1 Department of Nutrition, Faculty of Medicine and Health Sciences, Universiti Putra Malaysia, Serdang 43400, Selangor, Malaysia; muhamadhasrolmohdashri@gmail.com; 2 Department of Dietetics, Faculty of Medicine and Health Sciences, Universiti Putra Malaysia, Serdang 43400, Selangor, Malaysia; asyura@upm.edu.my; 3 Malaysian Research Institute of Ageing, (MyAgeing) Universiti Putra Malaysia, Serdang 43400, Selangor, Malaysia

**Keywords:** dietary supplements, sociodemographic factors, body weight status, dietary intake, government employees, Malaysia

## Abstract

The use of dietary supplements is prevalent among many groups worldwide. However, few studies have examined their use among government employees. The aim of this cross-sectional study was to determine the association among sociodemographic characteristics, body weight status, and energy intake with dietary supplement use among government employees in Putrajaya, Malaysia. Simple random sampling was used to select a sample of 460 government employees from six ministries in Putrajaya, Malaysia. The data used in this study were collected through anthropometric measurements (height, weight, % body fat, waist and hip circumferences), a self-administered questionnaire (sociodemographic characteristics and dietary supplements use), and an interviewer-administered questionnaire (24-hour dietary recall; fruit and vegetable intake). The results indicated that the prevalence of dietary supplement use was 55.4%, with vitamin C (38.4%) being the most popular type of dietary supplement. Health issues (80.8%) were the most common reason for usage, internet (59.2%) was the main source of information, and pharmacies (71.8%) were the most indicated places to purchase dietary supplements. A multivariate analysis showed that participants who were female, married, had better monthly income, lived within a smaller household size, had a normal body mass index, classified as having unhealthily high body fat percentage, did not skip breakfast, and consumed at least five servings of fruits and vegetables per day were significantly more likely to use dietary supplements. In conclusion, health-conscious groups were more prone to consume dietary supplements, and due to the high prevalence of dietary supplement use, dissemination of accurate scientific information regarding dietary supplements is highly recommended among government employees.

## 1. Introduction

Nowadays, the dietary supplement market is one of the world’s fastest-growing industries and continuously offers numerous products to the community. The United States Dietary Supplement Health and Education Act of 1994 defines dietary supplements as products that are taken by the mouth and are intended to supplement the diet by increasing the total energy and nutrient intake [1]. Dietary supplements contain one or more dietary ingredients, such as vitamins, minerals, amino acids, herbs and botanicals, and these products can be found in many forms, such as tablets, capsules, pills, liquids, and powders [1].

The prevalence of dietary supplement use has substantially risen over the past few years in both developed and developing countries. As seen in data compiled by the National Health and Nutrition Examination Survey (NHANES), the prevalence of dietary supplement use among US adults was 23% for 1971–1974, 35% for 1976–1980, 42% for 1988–1994, 52% for 1999–2000, and then remained relatively steady, with 54% for 2003–2006, 49% for 2007–2010, and 52% for 2011–2014 [2,3]. In Malaysia, in 2014, the Malaysian Adults Nutrition Survey (MANS) stated that the prevalence among the general adult population for use of multivitamin–mineral supplements and food supplements was 28% and 34%, respectively, which grew from 23.9% and 24.8%, respectively, compared to the previous MANS in 2003 [4].

This shows that there has been a high demand for dietary supplements, and these products have become acceptable to the public. Generally, dietary supplements have been massively advertised and commercialized as miraculous products that can contribute to many health benefits, and these over-the-counter products have also been widely available in various places, as they can be purchased without a prescription from a healthcare professional [5,6]. Nevertheless, several systematic reviews of randomized control trials have stated that there is still little and inconclusive evidence indicating the significant beneficial health effects of dietary supplements and that irrational use of these products might lead to several health risks [7,8]. In addition, the safety of using dietary supplements is still of concern, as these products may contain active ingredients, which could interact with medications or could contain other nutrients or excessive doses of potentially harmful ingredients and illegal substances that are not marked on the nutrition label [8]. Moreover, since dietary supplements have physiological and metabolic impacts on the human body, the decision to consume these products should be taken seriously and be clearly clarified based on an individual’s needs, such as increased physiological demands or an inability to meet necessary nutritional intake from solid foods [9]. Thus, advice or a prescription from a healthcare professional is recommended before using these products.

Nowadays, being overweight or obese is known as a public health problem worldwide in both developed and developing countries. In 2019, the National Health and Morbidity Survey (NHMS) stated that half of adults in Malaysia were overweight or obese, comprising 50.1% of the adult population [10]. The NHMS (2019) also indicated Putrajaya had the highest prevalence of overweight and the second highest state with obesity prevalence in Malaysia [10]. Putrajaya is the federal government center of Malaysia and its population comprised mainly government servants. Nutrition transitions, followed by work-related activity, and continuing to become more sedentary, influence body weight status, which increases the likelihood of the incidence of being overweight and obese in developing countries, including Malaysia [11,12]. Malaysia is experiencing a nutrition transition, where the population initially consumed a grains-based diet characterized by rich complex carbohydrates and fiber, which today has been exchanged for a diet high in meat, fat, oil and low in fiber [13]. Hence, these obesogenic factors lead adults to consider using dietary supplements in an effort to manage their body weight and diet [14]. 

The consumption of dietary supplements is well known among many groups of people, and government employees are of no exception [15]. As with other groups, government employees are also interested in improving their health to remain productive in the workplace, and they are likely to receive health benefits from dietary supplements. However, how sociodemographic, body weight status, and dietary intake contributes to dietary supplement use among government employees in Malaysia still remains undocumented, which means no studies have been conducted in this area. Hence, there is a need to understand which of these factors influence this group’s use of dietary supplements. Therefore, the aim of this study is to determine the association among sociodemographic characteristics, body weight status, and energy intake with dietary supplement use among government employees in Putrajaya, Malaysia. We hypothesized that dietary supplements use might related to sociodemographic characteristics, energy intake, and body weight status of the subjects.

## 2. Materials and Methods

### 2.1. Study Design and Participants

This cross-sectional study was conducted in the city of Putrajaya, Malaysia. Putrajaya is the federal government administrative center and is located 25 km from Kuala Lumpur, the national capital of Malaysia [16]. Simple random sampling was used to select a total of 460 government employees from six selected ministries to be the participants in this study. In random sampling, selected populations under study have an equal and independent chance of being included and considered the least biased method of sampling. The participants were eligible to participate in this study after meeting the study criteria, which were aged 18–59 years, male or female, Malaysian, permanent staff, able to understand the Malay or English language, not pregnant and absence of physical disability. An information sheet and consent form were provided to the participants, and data collection was conducted from August to November 2019. Permission from the head of secretaries in the selected ministries was obtained prior to commencement of the data collection. Additionally, the research protocol was approved by the Ethics Committee for Research Involving Human Subjects, reference no. UPM/TNCPI/RMC/1.4.18.2 (JKEUPM); 20 June 2019. 

### 2.2. Study Instruments

#### 2.2.1. Anthropometry Measurements

The anthropometric measurements included in this study were height, weight, body fat percentage, body mass index (BMI), waist and hip circumferences, and waist–hip ratio (WHR). Height was measured twice using a SECA 213 stadiometer, and the average was used for data analysis. The participants were required to take off their footwear and headgear, and were measured standing straight, head in the position of Frankfurt horizontal plane with their arms hanging loosely at their sides. Weight and body fat percentage were measured using a Tanita BC-418 body composition analyzer. To measure weight, the participants were required to empty their pockets, remove their shoes and socks, as well as take of any accessories that they wore on their wrist or hands. The data for height and weight were used to measure BMI through the formula weight divided by the square of height (kg/m^2^). The classifications for BMI were based on the World Health Organization (WHO) [17] and body fat percentage classifications were based on Lee and Nieman, respectively [18]. Furthermore, waist and hip circumferences were measured twice using a Lufkin W606 2M tape measure, and the average was used for data analysis. Waist circumference was measured at the midpoint between the lower margin of the last rib and the top of the iliac crest whereas hip circumference was measured around the widest portion of the buttocks. Waist circumference and WHR were classified based on the WHO [19].

#### 2.2.2. Self-Administered Questionnaire

For sociodemographic characteristics, participants were asked about their sex, ethnicity, marital status, and educational level through close-ended questions and asked about date of birth, monthly income, household income, household size, and job classification through open-ended questions. Participants was categorized into three groups based on monthly income adopted in Malaysia: the lowest 40% household income group; (≤RM 4849 per month), the 40% middle-income household group (RM 4850–RM 10,959 per month), and the highest/top income group of 20% households (at least RM 10,960 per month) [20].

In addition, participants were asked about their dietary supplement use through the 2014 NHMS adapted questionnaire [21]. The instrument used in the NHMS has been validated by the expert panel and undergone pre-tested [21]. First, the participants were asked whether they had used dietary supplements in the last 12 months through close-ended questions (‘yes’ or ‘no’). After that, the participants who answered that they had used dietary supplements were asked to complete the following questions regarding the types of supplements, frequency, reasons for usage, sources of information, places to purchase, and amount of money spent monthly on dietary supplements. In this section, the types of dietary supplements were classified into four categories: vitamin and/or mineral supplements, food supplements, nutritional ergogenic aids, and others. All types of dietary supplements assessed in this study were based on the definition from the Dietary Supplement Health and Education Act of 1994 [1]. For example, the ingredients, such as garlic, green tea, and honey, which have to go through the process of extraction, or a combination with other ingredients, and the end product can be found in a pill, powder, or a liquid, were noted as dietary supplements. The participants who were unsure about the type of dietary supplements used were asked to describe the dietary supplements, such as the packaging or label.

#### 2.2.3. Interviewer-Administered Questionnaire

Dietary intake was determined by using two days of 24-h dietary recall (one for weekday and one for weekend). The participants were required to recall all foods and beverages (excluding dietary supplements) consumed in detail, such as the name, amount consumed, time, location, ingredients, and methods of cooking. The serving sizes of the foods and drinks consumed were estimated by using household measurements, such as plates, bowls, spoons, glasses, and cups. Then, the dietary intake data were analyzed by using Nutritionist Pro Software to yield the data for the average energy and macronutrient intake. The percentage of energy adequacy was calculated based on the recommended nutrient intakes (RNI) energy recommendation for Malaysia [22]. Furthermore, the percentage of energy from carbohydrates, protein, and fat was classified based on the RNI [22]. Therefore, the present study followed the criteria as listed below to define acceptable energy and macronutrient intake, which is in line with the RNI and data analysis from the NHMS [21,22].

1. Energy intake should meet at least 80% of the energy recommendation by the RNI.
2. Carbohydrate intake should be in the range of 50–65% of total energy intake.
3. Protein intake should be in the range of 10–20% of total energy intake.
4. Fat intake should be in the range of 25–30% of total energy intake.

Furthermore, the participants were considered to have achieved the daily RNI recommended fruit and vegetable intake if they consumed five servings or above per day [22]. All forms of fruit and vegetable intake were identified, including raw, cooked, juiced, or dehydrated/dried.

### 2.3. Data Analysis

Statistical Package for the Social Sciences (SPSS) version 25 was used to analyze the collected data. Univariate analysis of the variables was applied to examine the distribution of the data and check for outliers. Regarding descriptive statistics, interval and ratio data were presented as mean and standard deviation. Meanwhile, nominal and ordinal data were presented as frequency and percentage. Associations between categorical variables were determined using the chi-square test. Furthermore, logistic regression analysis was performed to determine the significant contribution of predictors on dietary supplement use. Confidence intervals (CI) were set at 95% probability levels, and statistical significance was set at *p* < 0.05.

## 3. Results

In the present study, a total of 460 government employees in Putrajaya, Malaysia, were recruited, and the prevalence of dietary supplement users was 55.4% (Table 1). The majority of users consumed 1–2 types of dietary supplements (69.0%) more than once a week (35.7%), and had been using these products for 12 months or more (56.1%). The mean amount of money spent monthly on dietary supplements was reported by users as RM 116.67 ± 80.87.

Overall, vitamin C (38.4%) was the most popular type of dietary supplement used, followed by multivitamin–mineral (25.9), fish oil (22.7%), green tea (18.8%), and garlic pills (11.4%). There were multiple reasons reported for using dietary supplements, with the majority of users (80.8%) stating health reasons. As many as 35.7%, 29.4%, 25.5%, and 16.5% of users stated that their reasons for taking dietary supplements were to increase energy, for beautification, to lose fat, and to lose weight, respectively. In this study, users stated that the internet (59.2%) was the most popular source for obtaining information about dietary supplements. Other common sources were friends (49.0%), pharmacists (34.9%), and doctors (25.1%), while 20.0% said they self-prescribed. Moreover, participants identified several places of purchase for dietary supplements, with the majority pointing to pharmacies (71.8%), followed by supplement stores (38.0%) and online stores (24.3%).

Information on sociodemographic characteristics, body weight status, and dietary intake is shown in Table 2. The majority of dietary supplement users in this study were female (66.7%) and aged >30 years (78.4%). Most participants who consumed dietary supplements were Malay (82.4%), married (69.4), and possessed tertiary education (76.1%). The mean monthly income, household monthly income, and household size of dietary supplement users were RM 3667.82 ± 1494.78, RM 6232.32 ± 3193.34, and 1.52 ± 0.50 persons, respectively. 

Associations found through the chi-square test among sociodemographic characteristics, body weight status, and dietary intake with dietary supplement use among study participants are presented in Table 2. Regarding connections between dietary supplement use and sociodemographic characteristics—significant associations were found with sex (χ2 = 16.686; *p* ≤ 0.001), age (χ2 = 5.544; *p* = 0.019), ethnicity (χ2 = 6.827; *p* = 0.009), marital status (χ2 = 4.470; *p* = 0.034), educational level (χ2 = 3.918; *p* = 0.048), monthly income (χ2 = 13.316; *p* < 0.001), household monthly income (χ2 = 7.960; *p* = 0.019), household size (χ2 = 5.033; *p* = 0.025), and job classification (χ2 = 8.958; *p* = 0.003). Regarding body weight status, BMI (χ 2 = 4.440; *p* = 0.035) and body fat percentage (χ2 = 5.729; *p* = 0.017) were reported to be significantly associated with dietary supplement use. In addition, dietary supplement use had significant associations with percentage of energy from carbohydrates (χ2 = 10.838; *p* = 0.004) and fat (χ2 = 6.770; *p* = 0.034), breakfast consumption (χ2 = 17.711; *p* ≤ 0.001), and fruit and vegetable consumption (χ2 = 10.861; *p* = 0.001).

As shown in Table 3, multivariate logistic regression was performed to determine the contributions of various factors towards dietary supplement use. In this study, participants who were female (OR = 2.52; 95% CI = 1.59–3.97; *p* ≤ 0.001), had a smaller household size (OR = 2.33; 95% CI = 1.44–3.78; *p* = 0.001), had a normal BMI (OR = 2.06; 95% CI = 1.23–3.45; *p* = 0.006), were classified as having an unhealthily high body fat percentage (OR = 1.79; 95% CI = 1.05–3.09; *p* = 0.033), did not skip breakfast (OR = 3.12; 95% CI = 1.63–5.98; *p* = 0.001), and consumed at least five servings of fruits and vegetables (OR = 1.99; 95% CI = 1.29–3.10; *p* = 0.002) were significantly more likely to use dietary supplements.

## 4. Discussion

### 4.1. Prevalence and Distribution of Dietary Supplements

The present study found that more than half of the participants (55.4%) consumed dietary supplements. This prevalence was higher compared to findings from MANS, which reported that the percentage of the general adult population in Malaysia that consumes vitamin and mineral supplements and food supplements is 28.1% and 34.0%, respectively [4]. In addition, the prevalence of dietary supplement use in the current study was also higher than the finding from NHMS, which stated that 13.8% Malaysian adults use dietary supplements [10]. The rationale of this finding might be that government employees have an increased likelihood of being able to afford the cost of dietary supplements, as the government sector provides better financial compensation for its employees through its fixed salaries and allowances. In addition, in order to stay productive in the workplace, government employees might seek alternative health benefits from dietary supplements.

The prevalence of dietary supplement use in the present study is in line with other studies conducted among Malaysians, which have found supplement use to be 71.9% and 66.8%, respectively [23,24]. The prevalence of dietary supplement use in this study was also similar with previous studies in other countries. Recent findings from NHANES for 2011–2014 and the Council for Responsible Nutrition in 2019 stated that more than half of US adults consumed dietary supplements, with those percentages being 52.0% and 77.0%, respectively [2,25]. Furthermore, studies in Asian countries, such as Indonesia (63.0%), Korea (62.0%), and Saudi Arabia (55.3%), also reported that more than half of the participants used dietary supplements [26,27,28]. Specifically, the present study showed that the prevalence of dietary supplement use among government employees was higher compared to public secondary school teachers in Kenya (28.7%), university employees in Egypt (31.2%), and female health workers in Tehran (53.8%) [29,30,31].

Similar to findings from the MANS in 2014, and an earlier study in Malaysia, the present study found that the most popular type of dietary supplement among participants was vitamin C [4,23]. Vitamin C, or ascorbic acid, is a water-soluble vitamin and essential nutrient for the human body, which functions in the activation of enzymes, reduction of oxidative stress, and enhancement of immune function [32,33]. The most well-recognized health benefit of vitamin C is the prevention and treatment of the common cold [33]. However, the function of vitamin C related to the protection and relief of the common cold remains controversial [32]. Several clinical trials concluded that supplementing vitamin C in various doses had no significant prophylactic effect, but lessened the seriousness and length of cold symptoms throughout the infection cycle [32].

In this study, a high number of participants obtained information regarding dietary supplements through the internet, and this finding is in line with previous research [34]. This might be because, nowadays, the internet easily and quickly provides consumers with instant access to a great amount of information through the utilization of search engines. However, some internet sources have been noted as being highly questionable, which means information from this medium might be doubtful and inaccurate. Hence, this issue should raise concerns among healthcare professionals [28].

### 4.2. Factors Associated with Dietary Supplements

In relation to sociodemographic characteristics, multivariate analysis showed that females were significantly more likely to use dietary supplements, and this finding was similar with that of previous studies [31,35,36]. Females have been reported to be more aware of their health than males and, therefore, females might be more motivated to use dietary supplements to maintain and enhance their health [32]. In addition, previous research has also stated that participants from smaller households are significantly more likely to consume dietary supplements, which is consistent with the current study [36]. Those with a smaller household size are possibly able to reduce liabilities in terms of finances and increase financial resilience rates and, thus, this situation might contribute to enhancing their ability to cover the cost of dietary supplements.

Through bivariate analysis, the present study found that there was a significant association between age and dietary supplement use, with the majority of the older age group consuming dietary supplements. This finding was supported by previous studies [36,37,38]. This might be because, as one ages, they tend to become diagnosed with health problems, have increased concerns regarding health, develop the perception that their body is not getting enough nutrients, and gain the belief that dietary supplements are able to protect against diseases [31,37]. In addition, older people are more prone to have challenges in the intake, absorption, and metabolism of nutrients. Thus, the need for nutritional support through dietary supplements might be suggested. This study also revealed that marital status was significantly associated with dietary supplement use, with most married participants using dietary supplements [24]. A possible explanation could be due to a concern for the family’s health inspiring the consumption of dietary supplements among family members [24]. The findings from the present study are consistent with other studies, which showed that socioeconomic statuses, such as educational level, monthly income, household income, and job classification, were significantly associated with dietary supplement use [4,36,39]. It is known that a better educational level might result in better financial status and job position. People who possess a higher educational level are more prone to take care of their health, be more active in programs related to health, and are more likely to seek multiple sources of health-related knowledge [5]. In addition, a higher income also contributes to enhancing purchasing power and allows the individual to meet the costs involved with dietary supplement use. Hence, higher socioeconomic status might lead to increased use of dietary supplements.

Regarding body weight status, the multivariate analysis showed that BMI and body fat percentage were significant in predicting dietary supplement use, and these results were in line with others studies [14,40]. The current study confirmed that participants who had a normal BMI were more likely to use dietary supplements. However, the present study showed that participants who were classified as having an unhealthily high body fat percentage were more prevalent to consume dietary supplements. This is related to the thin outside, fat inside (TOFI) phenotype, which refers to those who have a relatively normal BMI but unconsciously have an alarming high level of fat in their body [41]. Nowadays, being overweight and obese is a severe worldwide public health concern [11,42]. In Malaysia, the NHMS reported that the prevalence of overweight and obese adults increased in 2006, 2011, and 2015 [43]. Overweight and obesity are important risk factors for numerous non-communicable diseases [11,42]. Since maintaining and achieving a normal range of body weight status is crucial for healthcare, participants might be motivated to seek alternative treatments through dietary supplements.

Moreover, in agreement with previous studies, this study also found that those who were health conscious, such as those who did not skip breakfast and those who consumed at least five servings of fruits and vegetables, were significantly more likely to use dietary supplements [44,45]. A previous study indicated that those who lived healthier lifestyles, which included consuming better diets, were more prone to taking dietary supplements [39]. Additionally, those who consumed dietary supplements were also noted to have higher levels of nutrient intake from solid foods alone. Therefore, they had a tendency towards lower nutrient inadequacy [39]. In truth, those who consume a well-balanced diet commonly can meet daily energy and nutrient requirements without supplementing with any products [9]. This could also prevent exceeding the tolerable upper intake levels of nutrients due to unjustified dietary supplementation, which can lead to nutrient toxicity [9].

According to a search of the literature, this is the first study to explore dietary supplement use and its associated factors specifically among government employees in Malaysia. The updated data from this study can contribute to intervention programs, future studies, references for healthcare professionals (especially dietitians and nutritionists), and evidence-based food and nutrition policies in Malaysia. Nevertheless, the design of this study was a cross-sectional study, which only determined the associations between the study variables. Hence, the findings of this study were unable to determine the causes, which contribute to dietary supplement use. Although the present study yielded the results on prevalence and associated factors of dietary supplement use, they were difficult to compare accurately with previous studies due to different definitions of dietary supplement users. Thus, this discrepancy suggests future research to provide guidelines for an exact definition of dietary supplement users, which can be used internationally. Furthermore, since the internet was the most popular source for obtaining information about dietary supplements reported in this study, healthcare professionals are recommended to maximize on this so they can spread reliable scientific information regarding dietary supplements through this medium, for example, by planning education and communication interventions related to this area. Considering the wide usage of dietary supplements, future research is recommended to increase the knowledge on psychological factors and nutrient adequacy in relation to dietary supplement use, in order to minimize unnecessary side effects and maximize the promotion of healthy dietary intake from solid foods. 

### 4.3. Strengths and Limitations of the Study

Several limitations in this study should be taken into consideration. First, the design of this study was cross-sectional, which only determined the relationship between study variables. Furthermore, a self-administered questionnaire might cause under- or over-reporting for certain sections. Although the present study yielded the results on prevalence of dietary supplements use and its associated factors, these results were somehow difficult to compare accurately with previous studies due to different or a variety of definitions of dietary supplement users.

Nevertheless, the main strengths of this study involved its interview techniques to gather data on dietary intake. This could minimize errors, such as over- and under-reporting in data collection. Random sampling was used to identify the participants to ensure an equal and independent chance of inclusion; moreover, it is considered the least biased method of sampling. The results of this study can provide updated data on prevalence and factors associated with dietary supplement use, which can be used as baseline information for future research or intervention programs in related fields.

## 5. Conclusions

In summary, there was a high prevalence rate of dietary supplement use among government employees in this study. A multivariate analysis showed that those who were more health conscious, such as females, those who had normal BMI, did not skip breakfast, and consumed at least five servings of fruits and vegetables were significantly more likely to use dietary supplements. In addition, participants who had economic advantages, including a smaller household size, and those who classified as having an unhealthily high body fat percentage, were more likely to consume dietary supplements. An appropriate dissemination of accurate information regarding dietary supplements is highly recommended among government employees. Hence, healthcare professionals, such as physicians, dietitians, nutritionists, and pharmacists, should combine their expertise to provide more comprehensive nutrition services to government employees by tackling the associated factors. Public education on the importance of using dietary supplements with the advice of and prescriptions from healthcare professionals should be strengthened.

## Figures and Tables

**Table 1:** Dietary supplements use among participants.

Dietary Supplements	Dietary Supplement Users *n* (%) Mean ± SD		
	Total (*n* = 255)	Male (*n* = 85)	Female (*n* = 170)
Dietary supplements use	255 (55.4)	85 (33.3)	170 (66.7)
Number of types of dietary supplements use	2.20 ± 1.10	2.33 ± 1.11	2.14 ± 1.09
1–2 3–4 ≥5	176 (69.0) 70 (27.5) 9 (3.5)	54 (63.5) 28 (32.9) 3 (3.5)	122 (71.8) 42 (24.7) 6 (3.5)
Frequency of using dietary supplements			
Everyday More than once a week Once a week 1–3 times per month Occasionally	84 (32.9) 91 (35.7) 33 (12.9) 35 (13.7) 12 (4.7)	22 (25.9) 33 (38.8) 11 (12.9) 17 (20.0) 2 (2.4)	62(36.5) 58(34.1) 22(12.9) 18(10.6) 10(5.9)
Duration has been using dietary supplements			
Less than one month One month and less than 12 months 12 months and above	24 (9.4) 88 (34.5) 143 (56.1)	7 (8.2) 23 (27.1) 55 (64.7)	17 (10.0) 65 (38.2) 88 (51.8)
Amount of monthly spent (RM)	116.67 ± 80.87	142.06 ± 96.81	103.98 ± 68.44
Vitamin–mineral supplements			
Vitamin C Multivitamin–mineral Calcium Vitamin E Magnesium Vitamin B complex Vitamin D Folic acid Vitamin B12	98 (38.4) 66 (25.9) 18 (7.1) 17 (6.7) 14 (5.5) 12 (4.7) 10 (3.9) 5 (2.0) 4 (1.6)	20 (23.5) 29 (34.1) 4 (4.7) 2 (2.4) 3 (3.5) 5 (5.9) 4 (4.7) 1 (1.2) -	78 (45.9) 37 (21.8) 14 (8.2) 15 (8.8) 11 (6.5) 7 (4.1) 6 (3.5) 4 (2.4) 4 (2.4)
Food supplements			
Fish oil Green tea Garlic pill Mix herbs Evening primrose oil Collagen Honey Tongkat ali Spirulina Kacip Fatimah Probiotic Gingko biloba Aloe vera	58 (22.7) 48 (18.8) 29 (11.4) 24 (9.4) 19 (7.5) 17 (6.7) 15 (5.9) 14 (5.5) 12 (4.7) 10 (3.9) 7 (2.7) 6 (2.4) 2 (0.8)	25 (29.4) 10 (11.8) 15 (17.6) 13 (15.2) - 1 (1.2) 10 (11.8) 14 (16.5) 5 (5.9) - 1 (1.2) 2 (2.4) -	33 (20.0) 38 (22.4 14 (8.2) 11 (6.5) 19 (11.2) 16 (9.4) 5 (2.9) - 7 (4.1) 10 (5.9) 6 (3.5) 4 (2.4) 2 (1.2)
Ergogenic aids			
Fat burner Whey protein Protein–carbohydrate Energy drinks/snacks Casein Creatine	19 (7.5) 18 (7.1) 10 (3.9) 7 (2.7) 4 (1.6) 3 (1.2)	10 (11.8) 11 (12.9) 4 (4.7) 5 (5.9) 4 (4.7) 2 (2.4)	9 (5.3) 7 (4.1) 6 (3.5) 2 (1.2) - 1 (0.6)
Reasons of usage			
Health Increased energy Beauty Lose fat Lose weight Healthcare prescription Gain muscle Improves memory Gain weight	206 (80.8) 91 (35.7) 75 (29.4) 65 (25.5) 42 (16.5) 38 (14.9) 15 (5.9) 8 (3.1) 5 (2.0)	66 (77.6) 45 (52.9) 6 (7.1) 16 (18.8) 13 (15.3) 15 (17.6) 10 (11.8) 3 (3.5) 3 (3.5)	140 (82.4) 46 (27.1) 69 (40.6) 49 (28.8) 29 (17.1) 23 (13.5) 5 (2.9) 5 (2.9) 2 (1.2)
Sources of information			
Internet Friend Pharmacist Doctor Self-prescribed Sale representative Family Books Nutritionist Fitness trainer Dietitian Television	151 (59.2) 125 (49.0) 89 (34.9) 67 (25.1) 51 (20.0) 42 (16.5) 39 (15.3) 29 (11.4) 29 (11.4) 28 (11.0) 19 (7.5) 16 (6.3)	42 (49.4) 51 (60.0) 26 (30.6) 24 (28.2) 16 (18.8) 17 (20.0) 15 (17.6) 6 (7.1) 12 (14.1) 14 (16.5) 6 (7.1) 5 (5.9)	109 (64.1) 74 (43.5) 63 (37.1) 40 (23.5) 35 (20.6) 25 (14.7) 24 (14.1) 23 (13.5) 17 (10.0) 14 (8.2) 13 (7.6) 11 (6.5)
Place to purchase			
Pharmacy Supplement store Online store Herbal medication store Supermarket Coach/trainer	183 (71.8) 97 (38.0) 62 (24.3) 16 (6.3) 10 (3.9) 8 (3.1)	54 (63.5) 37 (43.5) 26 (30.6) 11 (12.9) 3 (3.5) 4 (4.7)	129 (75.9) 60 (35.3) 36 (21.2) 5 (2.9) 7 (4.1) 4 (2.4)

**Table 2:** Associations among sociodemographic characteristics, body weight status and dietary intake with dietary supplements use among participants.

Factors	Dietary Supplements Use			
	Non-Users *n* (%)	Users *n* (%)	χ2	*p*-Value
Sex			16.686	<0.001 *
Male Female	108 (52.7) 97(47.3)	85 (33.3) 170 (66.7)		
Age (years)			5.544	0.019 *
<30 ≥30	65 (31.7) 140 (68.3)	55 (21.6) 200 (78.4)		
Ethnicity			6.827	0.009 *
Malay Non-Malay	187 (91.2) 18 (8.8)	210 (82.4) 45 (17.6)		
Marital Status			4.470	0.034 *
Single Married	83 (40.5) 122 (59.5)	78 (30.6) 177 (69.4)		
Educational level			3.918	0.048 *
Non-tertiary qualifications Tertiary	67 (32.7) 138 (67.3)	61 (23.9) 194 (76.1)		
Monthly income (RM)			13.316	<0.001 *
<3000 ≥3000	108 (52.7) 97 (47.3)	90 (35.3) 165 (64.7)		
Household income (RM)			7.960	<0.019 *
≤4849 4850–10,959 ≥10,960	99 (48.3) 96 (46.8) 10 (4.9)	92 (36.1) 141 (55.3) 22 (8.6)		
Household number (persons)			5.033	0.025 *
1–4 ≥5	99 (48.3) 106 (51.7)	151 (59.2) 104 (40.8)		
Job Classification			8.958	0.003 *
Support staff Officer	130 (63.4) 75 (36.6)	125 (49.0) 130 (51.0)		
BMI (kg/m^2^)			4.440	0.035 *
Normal Overweight and obese	93 (45.4) 112 (54.6)	142 (55.7) 113 (44.3)		
Body fat percentage (%)			5.729	0.017 *
Acceptable Unhealthy (high)	95 (46.3) 110 (53.7)	89 (34.9) 166 (65.1)		
Waist circumference (cm)			0.041	0.839
Acceptable Increased health risk	121 (59.0) 84 (41.0)	147 (57.6) 108 (42.4)		
Waist–hip ratio			0.001	0.975
Acceptable Increased health risk	125 (61.0) 80 (39.0)	154 (60.4) 101 (39.6)		
% of energy adequacy <80 ≥80	65(31.7) 140(68.3)	70(27.5) 185(72.5)	0.798	0.372
% of energy from carbohydrate			10.838	0.004 *
<50 50–65 >65	53 (25.9) 144 (70.2) 8 (3.9)	37 (14.5) 199 (78.0) 19 (7.5)		
% of energy from protein			0.000	1.000
10–20 >20	197 (96.1) 8 (3.9)	244 (95.7) 11 (4.3)		
% of energy from fat			6.770	0.034 *
<25 25–30 >30	29 (14.1) 62 (30.2) 114 (55.6)	59 (23.1) 78 (30.6) 118 (46.3)		
Skipping breakfast			17.711	<0.001 *
Yes No	44 (21.5) 161 (78.5)	19 (7.5) 236 (92.5)		
Fruits and vegetables (serving/d)			10.861	0.001 *
<5 ≥5	142 (69.3) 63 (30.7)	137 (53.7) 118 (46.3)		

* *p*-value < 0.05 represents significance. 1USD = RM4.2.

**Table 3:** Logistic regression analysis of various factors associated with dietary supplements use among participants.

Factors	OR	95% CI	*p*-Value
Sex			
Male Female	1 2.20	1.39–3.48	0.001 *
Age (years)			
<30 ≥30	1 1.32	0.72–2.43	0.369
Ethnicity			
Malay Non-Malay	1 1.78	0.93–3.41	0.083
Marital Status			
Single Married	1 1.48	0.83–2.65	0.188
Educational level			
Non-tertiary qualifications Tertiary	1 1.18	0.68–2.03	0.563
Monthly income (RM)			
<3000 ≥3000	1 1.60	0.93–2.77	0.092
Household income (RM)			
≤4849 4850–10,959 ≥10,960	1 1.33 1.66	0.68–2.60 0.52–5.33	0.397 0.396
Household size (persons)			
1–4 ≥5	2.26 1	1.37–3.73	0.001 *
Job classification			
Support staff Officer	1 1.59	0.94–2.69	0.087
BMI (kg/m^2^)			
Normal Overweight and obese	1.90 1	1.14–3.16	0.014 *
Body fat percentage (%)			
Acceptable Unhealthy (high)	1 1.76	1.03–3.02	0.039 *
% of energy adequacy <80 ≥80	1 1.23	0.82–1.84	0.319
% of energy from carbohydrate			
<50 50–65 >65	1 1.72 1.34	0.95–3.13 0.39–4.55	0.074 0.640
% of energy from fat			
<25 25–30 >30	1.66 1.01 1	0.82–3.33 0.60–1.70	0.157 0.965
Skipping breakfast			
Yes No	1 2.78	1.45–5.34	0.002 *
Fruits and vegetables (serving/day)			
<5 ≥5	1 1.85	1.19–2.88	0.007 *

* *p*-value < 0.05 represents significance.

## Data Availability

The data presented in this study are available on request from the corresponding author.
